# Surgeons' Exposure to Radiation in Single- and Multi-Level Minimally Invasive Transforaminal Lumbar Interbody Fusion; A Prospective Study

**DOI:** 10.1371/journal.pone.0095233

**Published:** 2014-04-15

**Authors:** Haruki Funao, Ken Ishii, Suketaka Momoshima, Akio Iwanami, Naobumi Hosogane, Kota Watanabe, Masaya Nakamura, Yoshiaki Toyama, Morio Matsumoto

**Affiliations:** 1 Department of Orthopaedic Surgery, Nerima General Hospital, Tokyo, Japan; 2 Department of Orthopaedic Surgery, School of Medicine, Keio University, Tokyo, Japan; 3 Department of Radiology, School of Medicine, Keio University, Tokyo, Japan; 4 Advanced Therapy for Spine and Spinal Cord Disorders, Keio University, Tokyo, Japan; 5 Society for Minimally invasive spine Stabilization (Clinical research group); University of Toronto, Canada

## Abstract

Although minimally invasive transforaminal lumbar interbody fusion (MIS-TLIF) has widely been developed in patients with lumbar diseases, surgeons risk exposure to fluoroscopic radiation. However, to date, there is no studies quantifying the effective dose during MIS-TLIF procedure, and the radiation dose distribution is still unclear. In this study, the surgeons' radiation doses at 5 places on the bodies were measured and the effective doses were assessed during 31 consecutive 1- to 3-level MIS-TLIF surgeries. The operating surgeon, assisting surgeon, and radiological technologist wore thermoluminescent dosimeter on the unshielded thyroid, chest, genitals, right middle finger, and on the chest beneath a lead apron. The doses at the lens and the effective doses were also calculated. Mean fluoroscopy times were 38.7, 53.1, and 58.5 seconds for 1, 2, or 3 fusion levels, respectively. The operating surgeon's mean exposures at the lens, thyroid, chest, genitals, finger, and the chest beneath the shield, respectively, were 0.07, 0.07, 0.09, 0.14, 0.32, and 0.05 mSv in 1-level MIS-TLIF; 0.07, 0.08, 0.09, 0.18, 0.34, and 0.05 mSv in 2-level; 0.08, 0.09, 0.14, 0.15, 0.36, and 0.06 mSv in 3-level; and 0.07, 0.08, 0.10, 0.15, 0.33, and 0.05 mSv in all cases. Mean dose at the operating surgeon's right finger was significantly higher than other measurements parts (P<0.001). The operating surgeon's effective doses (0.06, 0.06, and 0.07 mSv for 1, 2, and 3 fusion levels) were low, and didn't differ significantly from those of the assisting surgeon or radiological technologist. Revision MIS-TLIF was not associated with higher surgeons' radiation doses compared to primary MIS-TLIF. There were significantly higher surgeons' radiation doses in over-weight than in normal-weight patients. The surgeons' radiation exposure during MIS-TLIF was within the safe level by the International Commission on Radiological Protection's guidelines. The accumulated radiation exposure, especially to surgeon's hands, should be carefully monitored.

## Introduction

Open posterior lumbar interbody fusion (PLIF) [1] and transforaminal lumbar interbody fusion (TLIF) [2] have been widely developed in posterior spinal reconstruction surgery and demonstrated significant benefits to patients with various lumbar degenerative diseases. However, one of the drawbacks of both conventional open PLIF and TLIF procedures is the extensive soft tissue dissection and retraction of paraspinal muscles during the surgical approach, because the anatomic landmarks for pedicle screw insertion should be exposed [3], [4]. Minimally invasive lumbar spinal fusion using percutaneous pedicle screws was firstly described by Foley *et al* [5]. Khoo *et al* [6] also developed a minimally invasive technique in which PLIF is performed through a tubular retractor by combining a microendoscopic technique with percutaneous instrumentation. Recently, minimally invasive TLIF (MIS-TLIF), which reduces soft tissue dissections through a muscle-dilating approach, has widely developed and shown its advantages in the treatment of degenerative lumbar disease; it reduces blood loss, narcotic use, length of hospital stay, lost work time, and infection rates, and improves postoperative outcomes and cost-effectiveness [7]–[12]. MIS-TLIF has also shown its feasibility in revision settings [13], [14]. Selznick *et al*. reported revision MIS-TLIF was not associated with a higher rate of blood loss, infection or neurologic complication compared to primary MIS-TLIF.

Although MIS-TLIF is advantageous for patients with lumbar degenerative diseases, minimally invasive procedures performed under fluoroscopy have the disadvantage of exposing the surgical team to intraoperative radiation. Radiation doses accumulated by surgeons during MIS-TLIF should be precisely monitored and evaluated, to make sure the level of risk is appropriate. However, only a few studies have prospectively evaluated radiation exposure at multiple points during MIS-TLIF, therefore, the radiation dose distribution of the whole body was still unclear. Furthermore, there have been no studies quantifying the effective dose [15], which represents the radiation dose to the whole body that gives the same risk as the localized exposure, during MIS-TLIF procedure. The purpose of the present study was to quantify the radiation doses received by surgeons during single- and multi-level MIS-TLIF procedures. To the best of our knowledge, this is the largest prospective study investigating surgeons' intraoperative radiation exposure during 1- to 3-level MIS-TLIF.

## Materials and Methods

This study was approved by the institutional review boards of the participating institutions prior to beginning the study (Keio University, Nerima General Hospital, Tokyo, JAPAN). All patients gave the written informed consent before being included this study. Our study was conducted during 1- to 3-level MIS-TLIF surgeries for 31 consecutive patients, with a total of 46 levels of lumbar spinal fusion. The patients included 16 males and 15 females, with a mean age of 59.7±2.7 years (range 23–76). The mean body mass index (BMI) was 24.6±0.5 kg/m^2^ (range 20.3–31.6). MIS-TLIF was performed at 1 level in 20 patients (L2–3, 1; L3–4, 1; L4–5, 14; and L5–S1, 4), at 2 levels in 7 (L3–4–5, 5; L4–5–S1, 2), and at 3 levels in in 4 (L2–3–4–5, 1; L3–4–5–S1, 3). MIS-TLIF was performed on 12 patients with degenerative spondylolisthesis (DS), 6 with lumbar disc herniation (LDH) with Modic changes, 3 with degenerative lumbar scoliosis (DLS), 1 with lumbar facet cyst (LFC), and 9 with multiply-operated back (MOB) (Table 1). Operative indications for LDH with Modic change were severe low back pain which had poor responses to non-surgical treatments for more than 6 months, either Modic type 1 or type 2 change on magnetic resonance imaging, and temporal relief from a low back pain by an intradiscal anesthetic injection and/or a selective nerve root block at the affected level.

**Table 1:** A summary of cases in 1- to 3-level MIS-TLIF.

Case No.	Age	Sex	BMI (kg/m^2^)	Level	Disease	Surgical time (min)	Exposure time (min)
1	58	F	31.2	L4-5	DS	184	1.3
2	69	M	23.9	L4-5	MOB (LFC)	218	0.7
3	33	M	24.8	L5-S1	LDH	226	0.5
4	66	M	27	L5-S1	MOB (LDH)	176	0.8
5	75	F	21.8	L5-S1	MOB (ASD)	206	1.2
6	23	M	22.9	L4-5	LDH	153	0.5
7	75	F	22.5	L4-5	DS	190	0.9
8	63	F	23.1	L4-5	DS	240	0.9
9	27	M	21.8	L4-5	LDH	168	0.4
10	53	F	25.2	L4-5	LFC	155	0.3
11	69	F	24.2	L4-5	DS	169	0.4
12	37	M	24.8	L4-5	MOB (LDH)	135	0.4
13	61	F	25.1	L4-5	DS	160	0.5
14	74	F	24.9	L5-S1	DS	168	0.8
15	49	M	22.3	L4-5	LDH	148	0.4
16	33	M	24.9	L4-5	MOB (LDH)	144	0.4
17	62	F	21.1	L2-3	MOB (LDH)	114	0.5
18	56	M	24.5	L3-4	DS	121	0.4
19	73	M	24.6	L4-5	DS	120	0.5
20	58	F	20.3	L4-5	DS	242	1
21	63	M	24.9	L4-5-S1	LDH	249	1.2
22	64	F	20.5	L3-4-5	DS	343	1.6
23	51	F	30.5	L3-4-5	DS	259	0.7
24	61	M	31.6	L3-4-5	LDH	228	0.5
25	76	M	23.9	L3-4-5	MOB (LCS)	272	0.6
26	75	M	25.3	L4-5-S1	MOB (LCS)	271	0.7
27	74	F	26.1	L3-4-5	DS	236	0.9
28	69	F	27	L3-4-5-S1	DLS	411	0.9
29	76	M	22.8	L3-4-5-S1	DLS	263	0.9
30	63	M	23.7	L2-3-4-5	DLS	278	0.8
31	65	F	26.1	L3-4-5-S1	MOB (DLS)	290	1.3

(BMI; body mass index, DS; degenerative spondylolisthesis, LDH; lumbar disc herniation, DLS; degenerative lumbar scoliosis, LFC; lumbar facet cyst, ASD; adjacent segment disease, MOB; multiply-operated back).

**Figure 1:**
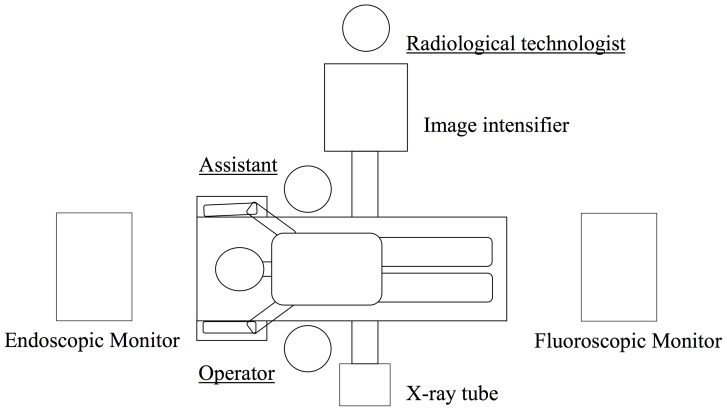
Intraoperative setup of the surgical team and equipment. A single C-arm was used for fluoroscopic imaging. The operating surgeon stood next to the X-ray tube. The assisting surgeon and radiological technologist were stationed at the opposite site, along with the image intensifier.

**Figure 2:**
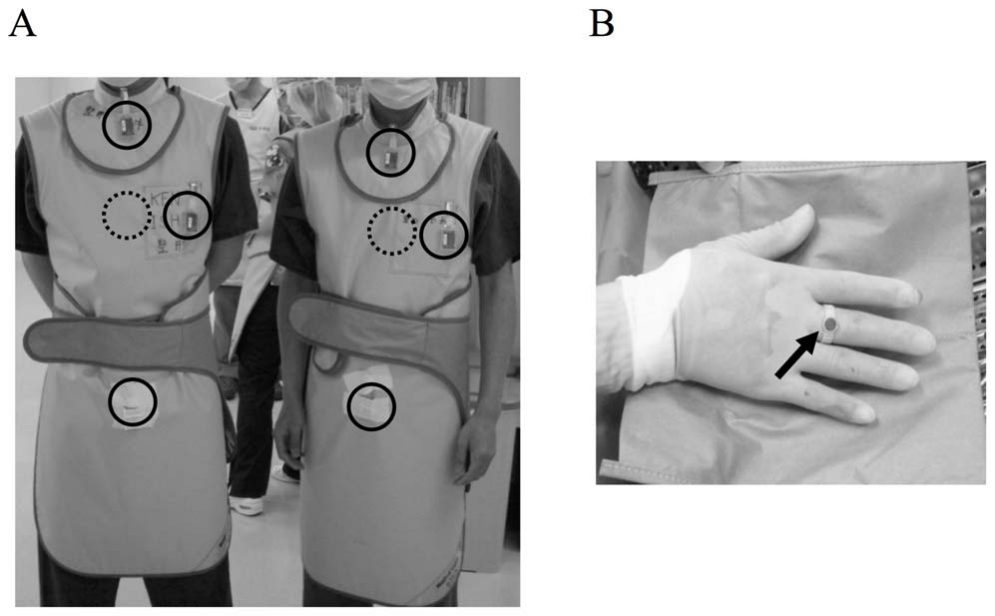
Placement of radiation monitors. The operating and assisting surgeon and radiological technologist wore thermoluminescent dosimeter (TLD) badges at the thyroid, chest, genitals (unshielded; circles in A), and on the chest (shielded; dotted circles in A). The operating and assisting surgeon wore sterile TLD ring badges on their right middle fingers (B).

All procedures were performed by a single operating surgeon (K.I) and a single assisting surgeon (H.F.). A single C-arm radiographic imaging unit (BV25 Gold6; Philips, Eindhoven, the Netherlands) equipped with 6-inch image intensifier system was used for fluoroscopic imaging. The operating surgeon stood beside the X-ray tube, and the assisting surgeon and radiological technologist stood on the opposite site (Figure 1). We wore a lead apron (Hagoromo 0.25 mm Pb; Maeda Co., Japan) that covers the entire torso, and a thyroid collar to protect all of the organs except head. The operating and assisting surgeons and the radiological technologist wore thermoluminescent dosimeter (TLD) badges (Sangyo Kagaku, Co., Japan) at unshielded points—the thyroid, chest, and genitals—and on the chest under a lead apron (Figure 2A). The operating and assisting surgeons wore sterile TLD ring badges on their right hands (middle finger) (Figure 2B). Measurement range of the TLD badges was 0.01 to 10000 mSv. Radiation doses (mSv) were measured from the TLD badges for each MIS-TLIF procedure. The dose at the lens of the eye was calculated as the thyroid dose ×0.905 (conversion factor; 1-cm depth to 0.07-cm depth, 70 keV). The effective dose, defined by the International Commission on Radiation Units and Measurements Inc. (ICRU) [15], was calculated as follows: *E* = 0.11 *Ha* +0.89 *Hb*, where *Ha* was the dose measured at the thyroid (1-cm depth), and *Hb* was the dose measured on the chest under the apron (1-cm depth). We also recorded the fluoroscopic exposure times and the outputs in kVp and mA.

**Figure 3:**
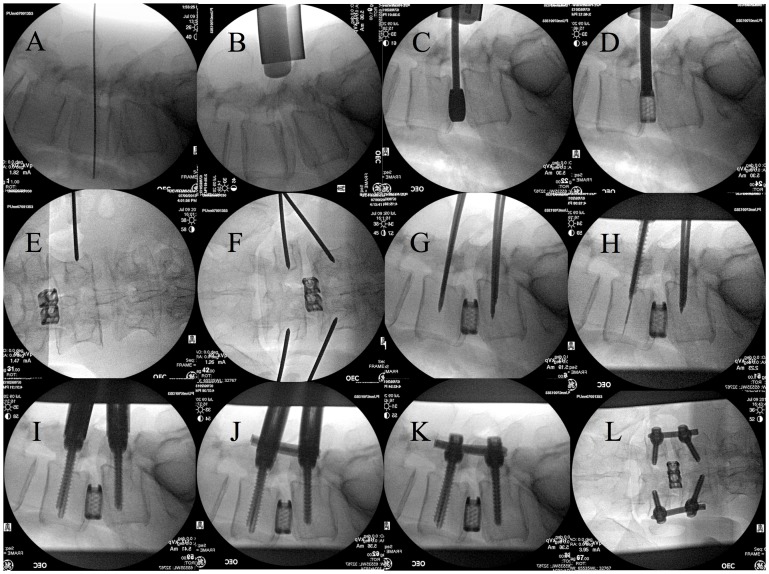
Utilization of fluoroscopy. Fluoroscopy was typically involved at 5 steps during MIS-TLIF: preoperative skin marking (A), confirmation of the retractor position (B), placement of the cage (C, D), insertion of the percutaneous pedicle screws (E–I), and placement of the rods (J–L). We used one-shot imaging, which uses short exposure times rather than continuous exposure.

**Figure 4:**
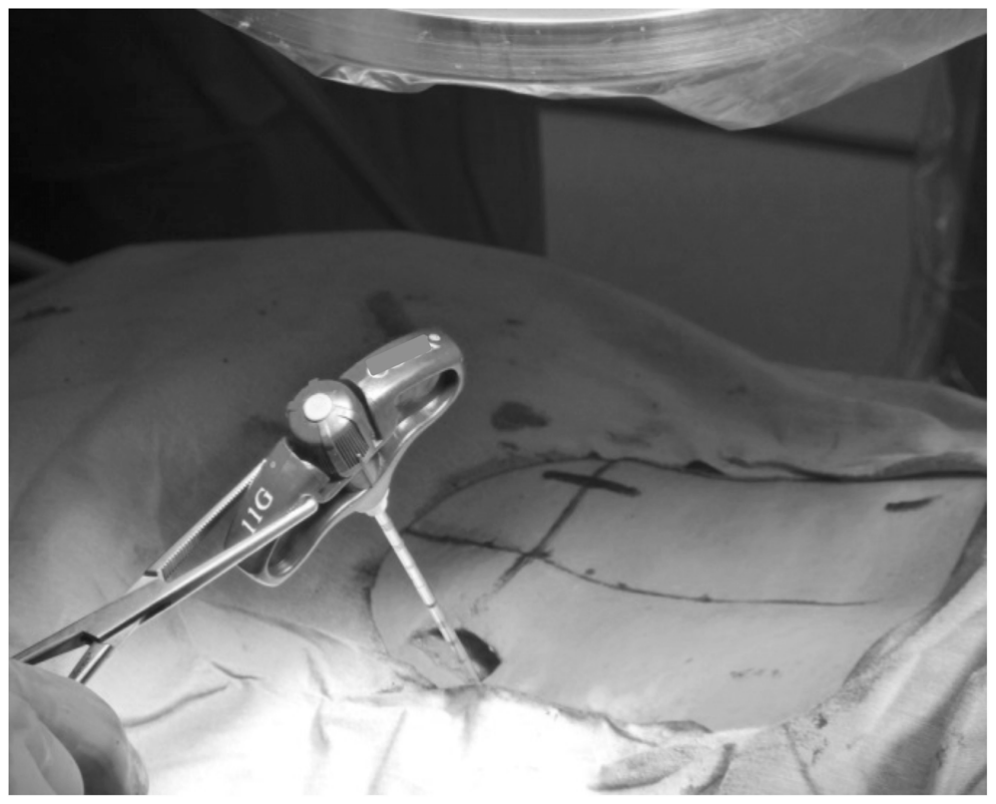
Protective techniques against excessive radiation exposures. Surgeons should always pay attention to the location of their hands at every fluoroscopic shots during MIS-TLIF procedures. The hands are often exposed to the X-ray beam while confirming the insertion point of the Jamshidi needle. A long Kocher clamp can be used to hold the Jamshidi needle for keeping a distance from the X-ray tube.

Fluoroscopy was typically involved at 5 steps during MIS-TLIF: preoperative skin marking (Figure 3A), confirmation of the retractor position (Figure 3B), placement of the cage (Figure 3C and D), insertion of the percutaneous pedicle screws (Figure 3E–I), and placement of the rods (Figure 3J–L). We used one-shot imaging, which provides short exposure times rather than continuous exposure. During the insertion of the percutaneous pedicle screws, we held the Jamshidi needle with a long Kocher clamp to distance the hands from the X-ray tube (Figure 4).

One-way ANOVA followed by Fisher's PLSD post-hoc test were used for statistical comparison in surgical time, estimated blood loss (EBL), and fluoroscopic exposure time between 1-, 2-, and 3-level MIS-TLIF, and in radiation doses between the operating surgeon, assisting surgeon, and radiological technologist. Student *t* test were used to compare surgical time, EBL, fluoroscopic exposure time, and radiation doses between primary and revision surgery, and between normal- and over-weight patients. SPSS software (14.0 J, SPSS Japan Inc., Tokyo, Japan) was used for analyses, and a P value less than 0.05 was considered significant. All data were expressed as the mean ± standard error.

## Results

**Figure 5:**
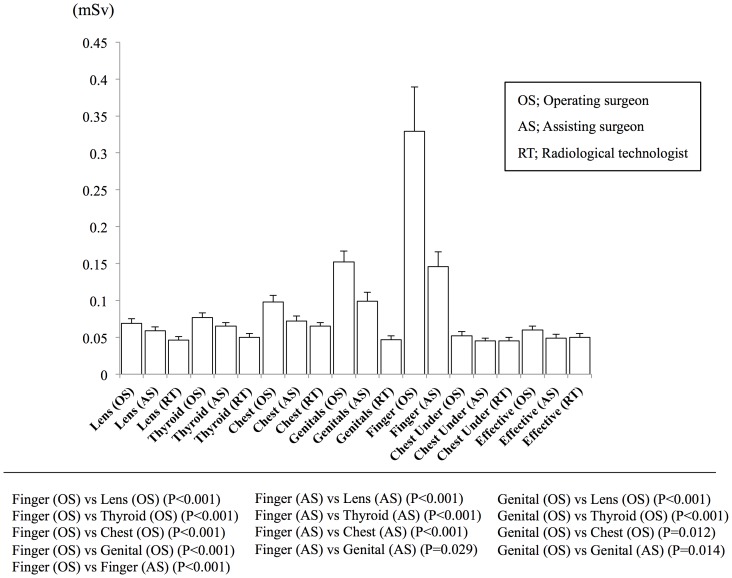
The mean radiation doses measured at different areas, and mean effective doses in all cases. The mean radiation doses and effective doses are shown for all cases. The mean radiation doses measured at the operating and assisting surgeons' right middle fingers were significantly higher than those measured elsewhere on the bodies. The mean radiation dose measured at the operating surgeon's genital area was significantly higher than at the lens, thyroid, or chest. Mean effective doses didn't differ significantly among the operating surgeon, assisting surgeon, and radiological technologist.

The mean fluoroscopic exposure times during 1-, 2-, or 3-level MIS-TLIF, respectively, were 38.7±17.1, 53.1±23.3, and 58.5±13.3 seconds. The fluoroscopic exposure time tended to be longer in 2-level than in 1-level MIS-TLIF (P = 0.092). The mean fluoroscopic exposure time was significantly longer in 3-level than in 1-level MIS-TLIF (P = 0.041). The tube voltage and current were automatically controlled with average readings 99.9±1.1 kVp and 3.3±0.2 mA, respectively. Table 2 shows the mean radiation and effective doses for the operating surgeon, assisting surgeon, and radiological technologist (P values were shown in Figure 5). For all cases, the mean radiation dose at the right middle finger was 0.33±0.06 mSv for the operating surgeon and 0.15±0.02 mSv for the assisting surgeon; for the operating surgeon, this dose was significantly higher than the doses measured elsewhere on the body (P<0.001), and significantly higher than the corresponding dose measured for the assisting surgeon (P<0.001). The assisting surgeon's mean dose at the right finger was also significantly higher than doses at the lens (P<0.001), thyroid (P<0.001), chest (P<0.001), or genitals (P = 0.029). Interestingly, the operating surgeon's mean radiation dose at the genitals was 0.15±0.01 mSv, which was significantly higher than doses at the lens (P<0.001), thyroid (P<0.001), and chest (P = 0.012); this dose was also significantly higher for the operating surgeon than for the assisting surgeon (P = 0.014) The mean effective doses for the surgeon performing 1-, 2-, or 3-level MIS-TLIF (0.06±0.01, 0.06±0.01, and 0.07±0.02 mSv) were not significantly different from those received by the assisting surgeon (0.04±0.01, 0.06±0.01, and 0.07±0.02 mSv) or the radiological technologist (0.05±0.01, 0.06±0.01, 0.06±0.02 mSv).

**Table 2:** Mean surgeons' radiation doses of different measurement parts, and mean effective doses in MIS-TLIF (mSv).

	Overall (n = 31)			1-level (n = 20)			2-level (n = 7)			3-level (n = 4)		
	OS	AS	RT	OS	AS	RT	OS	AS	RT	OS	AS	RT
Crystal lens	0.07±0.01	0.06±0.01	0.05±0.01	0.07±0.01	0.05±0.01	0.04±0.01	0.07±0.01	0.07±0.01	0.05±0.01	0.08±0.02	0.07±0.02	0.06±0.01
Thyroid	0.08±0.01	0.07±0.01	0.05±0.01	0.07±0.01	0.06±0.01	0.05±0.01	0.08±0.01	0.07±0.01	0.05±0.01	0.09±0.02	0.08±0.02	0.06±0.02
Chest 1	0.10±0.01	0.07±0.01	0.07±0.01	0.09±0.01	0.06±0.01	0.06±0.01	0.09±0.02	0.09±0.02	0.07±0.02	0.14±0.04	0.09±0.01	0.07±0.01
Genitals	* 0.15±0.01	0.10±0.02	0.05±0.01	* 0.14±0.01	0.08±0.01	0.05±0.01	* 0.18±0.03	0.15±0.05	0.05±0.01	0.15±0.05	0.13±0.04	0.05±0.01
Finger	* 0.33±0.06	* 0.15±0.02		* 0.32±0.08	* 0.14±0.03		* 0.34±0.10	0.14±0.04		* 0.36±0.10	* 0.20±0.02	
Chest 2	0.05±0.01	0.05±0.01	0.05±0.01	0.05±0.01	0.04±0.01	0.04±0.01	0.05±0.01	0.05±0.01	0.05±0.01	0.06±0.02	0.06±0.02	0.05±0.01
Effective dose	0.06±0.01	0.05±0.01	0.05±0.01	0.06±0.01	0.04±0.01	0.05±0.01	0.06±0.01	0.06±0.01	0.06±0.01	0.07±0.02	0.07±0.02	0.06±0.02

(OS; operating surgeon, AS; assisting surgeon, RT; radiological technologist. Chest 1; a dose at unshielded chest, Chest 2; a dose at chest under a lead apron. *Statistically significant).

The mean surgical time was 171.9±8.6 minutes in 1-level MIS-TLIF, 265.4±14.4 minutes in 2-level MIS-TLIF, and 310.5±34.0 minutes in 3-level MIS-TLIF. There were statistically significant differences in the mean surgical times for single and multi-level MIS-TLIF (1-level *vs*. 2- or 3-level; P<0.001). The mean EBL were 178.2±24.1 ml in 1-level MIS-TLIF, 416.1±108.2 ml in 2-level MIS-TLIF, and 360.5±56.4 ml in 3-level MIS-TLIF. There were statistically significant differences in the mean estimated blood loss for single and multi-level MIS-TLIF (1-level *vs*. 2-level; P = 0.003, 1-level *vs*. 3-level; P = 0.006). There were no significant differences in surgical time and EBL between 2- and 3-level MIS-TLIF. No major complications were observed during the perioperative period for any patient.

Because our case series included only 2 patients in 2-level MIS-TLIF and 1 in 3-level who had a revision surgery, we compared radiation doses between primary and revision surgery in 1-level MIS-TLIF (primary; n = 14, revision; n = 6). There were no significant differences in surgical time (primary; 174.6±10.4 *vs*. revision; 165.5±16.9 minutes), EBL (primary; 191.2±33.4 *vs*. revision; 147.7±17.6 ml), and fluoroscopic exposure time (primary; 38.1±4.6 *vs*. revision; 40.0±7.5 seconds) between the two groups. There were no significant differences in radiation doses at all measurement parts between primary and revision surgery (Table 3).

**Table 3:** Comparison of surgeons' radiation doses between primary and revision surgery in 1-level MIS-TLIF.

	OS			AS			RT		
	Primary (n = 14)	Revision (n = 6)	P	Primary (n = 14)	Revision (n = 6)	P	Primary (n = 14)	Revision (n = 6)	P
Crystal lens	0.07±0.01	0.07±0.01	0.972	0.05±0.01	0.06±0.01	0.952	0.05±0.01	0.04±0.01	0.843
Thyroid	0.07±0.01	0.08±0.02	0.845	0.06±0.01	0.06±0.01	0.912	0.05±0.01	0.04±0.01	0.875
Chest 1	0.09±0.01	0.09±0.02	0.924	0.06±0.01	0.07±0.02	0.912	0.06±0.01	0.07±0.01	0.758
Genitals	0.14±0.02	0.16±0.03	0.717	0.07±0.01	0.09±0.03	0.687	0.05±0.01	0.05±0.01	0.966
Finger	0.32±0.10	0.33±0.16	0.759	0.15±0.04	0.10±0.03	0.317			
Chest 2	0.05±0.01	0.06±0.02	0.759	0.04±0.01	0.04±0.01	0.960	0.04±0.01	0.05±0.02	0.889
Effective dose	0.06±0.01	0.06±0.01	0.980	0.04±0.01	0.04±0.01	0.996	0.04±0.01	0.05±0.01	0.885

(OS; operating surgeon, AS; assisting surgeon, RT; radiological technologist. Chest 1; a dose at unshielded chest, Chest 2; a dose at chest under a lead apron.).

Normal-weight patients (BMI<25, n = 16) and over-weight patients (BMI>25, n = 4) were also compared in 1-level MIS-TLIF. There were no significant differences in surgical time (BMI<25; 172.6±10.8 *vs*. BMI>25; 168.8±6.8 minutes), EBL (BMI<25; 194.3±28.1 *vs*. BMI>25; 113.5±27.8 ml), and fluoroscopic exposure time (BMI<25; 37.5±3.8 *vs*. BMI>25; 43.5±13.0 seconds) between the two groups. The operating surgeon's mean dose at the chest was significantly higher in over-weight patients (0.14±0.03 mSv) compared to normal-weight patients (0.08±0.01 mSv) (P = 0.002). And, the assisting surgeon's mean dose at the genitals was significantly higher in over-weight patients (0.12±0.04 mSv) compared to normal-weight patients (0.06±0.01 mSv) (P = 0.039). There were no significant differences in radiation doses at other measurement parts between the two groups (Table 4).

**Table 4:** Comparison of surgeons' radiation doses between normal-weight (BMI<25) and over-weight patients (BMI>25) in 1-level MIS-TLIF.

	OS			AS			RT		
	BMI<25 (n = 16)	BMI>25 (n = 4)	P	BMI<25 (n = 16)	BMI>25 (n = 4)	P	BMI<25 (n = 16)	BMI>25 (n = 4)	P
Crystal lens	0.06±0.01	0.08±0.02	0.198	0.05±0.01	0.08±0.02	0.053	0.05±0.01	0.03±0.01	0.106
Thyroid	0.07±0.01	0.09±0.02	0.288	0.05±0.01	0.08±0.02	0.066	0.05±0.01	0.03±0.01	0.142
Chest 1	0.08±0.01	*0.14±0.03	0.002	0.06±0.01	0.09±0.03	0.095	0.07±0.01	0.05±0.02	0.253
Genitals	0.15±0.02	0.13±0.02	0.623	0.06±0.01	*0.12 ±0.04	0.039	0.05±0.01	0.04±0.02	0.714
Finger	0.32±0.11	0.33±0.06	0.963	0.13±0.03	0.18±0.08	0.722			
Chest 2	0.05±0.01	0.07±0.01	0.225	0.04±0.01	0.06±0.02	0.185	0.05±0.01	0.02±0.00	0.136
Effective dose	0.05±0.01	0.07±0.01	0.170	0.04±0.01	0.06±0.02	0.057	0.05±0.01	0.03±0.01	0.103

(OS; operating surgeon, AS; assisting surgeon, RT; radiological technologist. Chest 1; a dose at unshielded chest, Chest 2; a dose at chest under a lead apron. *Statistically significant).

## Discussion

While studies have addressed intraoperative radiation exposures during orthopedic surgeries, the effective doses and doses received at different parts of the body in single- and multi-level MIS-TLIF have not been studied, and the surgeon's intraoperative radiation exposure has not been clarified. In our prospective case-series study, radiation doses at 5 places on the bodies were measured and the effective doses were assessed for the operating surgeon, assisting surgeon, and radiological technologist during 31 consecutive 1- to 3-level MIS-TLIF surgeries. In previous studies reporting that surgeons are exposed to radiation during MIS-TLIF, dosimeters were not worn both inside and outside the protective apron [16]–[18], and the effective dose was not investigated despite its importance [19]. The surgeon's exposure cannot be accurately gauged without measuring the dose inside the apron and investigating the effective dose. Järvinen *et al* [20] emphasized the usage of two dosimeters, placed outside and underneath the apron. The ICRP also recommends that personnel with a high risk of exposure during interventional radiology use two dosimeters [21], [22]; usually, one dosimeter is worn on the chest inside the apron, and the other placed on the neck, outside the apron. The effective dose [15], which is calculated from doses measured both inside and outside the apron, is useful for accurately evaluating occupational limits. However, few studies have investigated the effective dose of fluoroscopic radiation during spine surgeries [23], and there have been no studies quantifying the effective dose during MIS-TLIF procedure. Guidelines from the 1990 International Commission on Radiological Protection (ICRP) allow occupational radiation exposure to accumulate to a maximum annual radiation exposure of 150 mSv to the crystal lens, 500 mSv to the skin or hands, and 20 mSv as the effective dose [21]. In our study, the mean effective doses for the operating surgeon, the assistant, and the radiological technologist of 0.06, 0.05, and 0.05 mSv, respectively. The mean effective dose received by the operating surgeon was well within the safe level, equivalent to 0.3% of the annual occupational limit. One of the reasons for the low radiation exposure is that we typically use a one-shot rather than continuous fluoroscopy exposure technique. Indeed, Goodman *et al* [24] reported pulsed and low-dose fluoroscopy modes reduced exposure times by 56.7% in spinal interventional procedures (e.g. facet injection, lumbar sympathetic block, radiofrequency ablation). In a cadevaric study, Rampersaud *et al* [25] reported a mean dose at the neck of 8.3 mrem (0.083 mSv)/minute, and a mean hand dose of 58.2 mrem (0.582 mSv)/minute during assisted pedicle-screw insertion in spine surgery. Bindal *et al* [19] reported a mean fluoroscopy time of 1.69 minutes per case during 1- or 2-level MIS-TLIF, and a mean exposure to the surgeon per case of 76 mrem (0.76 mSv) to the surgeon's dominant hand, 27 mrem (0.27 mSv) at the waist under a lead apron, and 32 mrem (0.32 mSv) at the level of the unprotected thyroid. In our study, the surgeon's average of the doses measured at the 5 different points were much lower than those reported previously. Therefore, our one-shot fluoroscopic technique would be effective for reduction of radiation exposure.

However, the averaged doses of radiation to the right middle finger of the operating surgeon and assisting surgeon were 0.33 and 0.15 mSv, which were significantly higher than the doses measured at other unshielded areas of the bodies. Previous studies have reported a dose range from 0.08 and 0.2 mSv to the surgeon's hands during routine orthopaedic procedures [26]–[28]. In femoral or tibial intramedullary nailing, doses of 1.2–1.3 mGy per procedure to the hands have been reported [29]. Jones *et al* [30] reported that in lumbar spine surgery, the surgeon's hands receive a dose of 0.9 mGy per procedure for pedicle screw insertions. The ICRP and the National Radiological Protection Board demonstrated the threshold radiation doses for damage to the skin [21], [22], [31], [32]. Ionizing radiation can cause both acute and chronic skin damage. Although transient erythema caused by acute skin radiation exposure persists for 3 to 9 weeks, repetitive fluoroscopy procedures can also lead to chronic skin damage. Chronic radiodermatitis, which can present from months to several years after irradiation, causes dermal atrophy and telangiectasia. Chronic skin injuries resulting from interventional cardiology procedures have been reported [33]. Moreover, accumulated radiation exposure to the skin is a concern because of the potential of carcinogenesis. Radiation exposure can damage DNA, with cytotoxic and carcinogenic effects [34]. Shan *et al* [35] reported a patient with multiple syringoid eccrine carcinomas in both hands after long-term occupational exposure to X-rays. Radiation exposure to the hands is a long-standing problem; the surgeon or technologist often has to be close to both the X-ray beam and the patient to carry out the procedure [36]. In the orthopedic field, surgeons must maintain the location of instruments under the X-ray beam, and face a risk of high radiation exposure. According to the literature, maintaining a 5–10 cm distance from the patient can reduce exposure by 25–45% [25]. Surgeons should always pay attention to the location of their hands at every fluoroscopic shots during MIS-TLIF procedure. During placement of the percutaneous pedicle screws, surgeons must confirm the position of the Jamshidi needle and pedicle screw under fluoroscopy. At this step, the surgeon's hands often have a high risk of exposure to the X-ray beam. Therefore, we strongly recommend holding the Jamshidi needle with a long Kocher clamp during the fluoroscopic shots to distance the hands from the X-ray tube (Figure 4).

Wearing a lead apron and a thyroid collar is reported to reduce the effective dose [37], [38]. Interestingly, our study showed that the mean radiation dose at the genitals was significantly higher than that measured at any other point except the surgeon's hand. We ascertain that the genital area is closer to the X-ray tube (especially in lateral shots) and to the patients, and thus that the genitals are more likely to receive both direct and scattered radiation. Therefore, it is also important to wear a lead apron that covers the entire torso.

In this study, primary and revision surgery were compared in 1-level MIS-TLIF. Our study showed no significant differences in surgical time, EBL, and fluoroscopic exposure time between the two groups. Selznick *et al*. [13] reported blood loss was slightly higher in revision MIS-TLIF, however, it was not significantly high. They also found there was no significant higher rate of infection or neurologic complication between primary and revision MIS-TLIF. Our results also showed there were no significant differences in radiation doses at all measurement parts between primary and revision MIS-TLIF. Wang *et al*. [14] reported their 52 revision TLIF case series, and they found the mean fluoroscopic exposure time in revision MIS-TLIF (72 seconds) was significantly longer than that in revision open-TLIF (39 seconds). Our results showed the mean fluoroscopic exposure time in revision MIS-TLIF was 44.0 seconds. Therefore, one-shot fluoroscopic technique was considered as a key factor for reduction of fluoroscopic exposure time. In this study, most of MOB patients already had previous decompression surgery and they showed one-side lateral recess stenosis or intervertebral instability. For this reason, decompression and interbody fusion were completely performed from one-side lateral approach. Because we didn't have to remove all the scar tissues from the dura when we decompressed a nerve root and approached to the disc, it seemed not to be related to a time-consuming procedure due to previous surgeries.

We also compared normal- and over-weight patients. The operating surgeon's mean dose at the chest and the assisting surgeon's mean dose at the genitals were significantly higher, and the fluoroscopy also tended to have a longer exposure time in over-weight patients. It is sometimes difficult to get a good fluoroscopic resolution, and the tube voltage and current are automatically adjusted for signal-to-noise in over-weight patients. Kuon *et al*. [39] reported that higher BMI and larger body surface area were associated with higher radiation exposures in their invasive cardiac procedures. Ector *et al*. [40] also found that BMI was more determinant of radiation dose than a total fluoroscopic exposure time in their catheter ablation case series. Although there were no significant differences in surgical time, EBL, and fluoroscopic exposure time between normal- and over-weight patients in our study, higher scatter radiation caused by higher radiation outputs and larger body surface areas might increase the surgeons' radiation doses in over-weight patients. Ideally, surgeons should distance their bodies from the X-ray tube and the patient during a floroscopy shot.

Our study found considerably less intraoperative radiation exposure than has previously been reported [16], [17], [19]. Our findings suggested that careful fluoroscopy techniques, such as avoiding continuous exposure and keeping a distance from the X-ray tube, can effectively reduce radiation exposure times and doses. Our data emphasize the importance of taking basic precautions against excessive radiation exposure. Additionally, several authors reported that protective gloves [41], [42] and a navigation system [17] may also reduce radiation exposure. Our goal on the development of MIS-TLIF is to seek how to carry out procedures safely with low radiation exposure or without any radiation exposures. In this study, we demonstrated the importance of taking basic precautions during MIS-TLIF procedure. We also expect that either new lower-radiation alternative or protective equipment would be developed for MIS-TLIF near future.

## Conclusions

The present study demonstrated that radiation doses to surgeons during single- and multi-level MIS-TLIF were well within the level of safe occupational exposure risk outlined by ICRP 1990. Appropriate fluoroscopy techniques, such as avoiding continuous fluoroscopy and keeping an adequate distance from the X-ray tube, can effectively reduce excessive radiation exposure. Revision MIS-TLIF was not associated with higher surgeons' radiation doses compared to primary MIS-TLIF. Surgeons' radiation doses were higher in over-weight patients than in normal-weight patient during MIS-TLIF procedure. The accumulated radiation exposure, especially to the surgeon's hands, should be carefully monitored.
